# *In situ* stable isotope probing of phosphate-solubilizing bacteria in the hyphosphere

**DOI:** 10.1093/jxb/erv561

**Published:** 2016-01-21

**Authors:** Fei Wang, Ning Shi, Rongfeng Jiang, Fusuo Zhang, Gu Feng

**Affiliations:** College of Resources and Environmental Sciences, China Agricultural University, Beijing 100193, PR China

**Keywords:** AM fungus, ^13^CO_2_ pulse labeling, hyphosphere, maize, organic phosphate, phosphate-solubilizing bacteria (PSB).

## Abstract

This study used a [^13^C]DNA stable isotope probing (SIP) technique to elucidate a direct pathway for the translocation of ^13^C-labeled photoassimilate from maize plants to extraradical mycelium-associated phosphate-solubilizing bacteria (PSB) that mediate the mineralization and turnover of soil organic phosphorus (P) in the hyphosphere. Inoculation with PSB alone did not provide any benefit to maize plants but utilized the added phytate-P to their own advantage, while inoculation with *Rhizophagus irregularis* alone significantly promoted shoot biomass and P content compared with the control. However, compared with both sole inoculation treatments, combined inoculation with PSB and *R. irregularis* in the hyphosphere enhanced organic P mineralization and increased microbial biomass P in the soil. There was no extra benefit to plant P uptake but the hyphal growth of *R. irregularis* was reduced, suggesting that PSB benefited from the arbuscular mycorrhizal (AM) fungal mycelium and competed for soil P with the fungus. The combination of T-RFLP (terminal restriction fragment length polymorphism) analysis with a clone library revealed that one of the bacteria that actively assimilated carbon derived from pulse-labeled maize plants was *Pseudomonas alcaligenes* (Pseudomonadaceae) that was initially inoculated into the hyphosphere soil. These results provide the first *in situ* demonstration of the pathway underlying the carbon flux from plants to the AM mycelium-associated PSB, and the PSB assimilated the photosynthates exuded by the fungus and promoted mineralization and turnover of organic P in the soil.

## Highlight

Phosphate-solubilizing bacteria associated with extraradical mycelia of *Rhizophagus irregularis* in the hyphosphere assimilated ^13^C-labeled plant photosynthates and enhanced the mineralization and turnover of soil organic P.

## Introduction

Phosphorus (P) security is emerging as one of the greatest global sustainability challenges of the 21st century (Cordell and White, 2014). Organic phosphates account for 20–80% of the total P in a wide range of soils (Dalal, 1977) and contribute substantially to the P nutrition of plants. Soil microorganisms play a key role in the major biogeochemical nutrient cycles, including those of organic carbon (C), nitrogen (N) and phosphorus (P), and in the control of nutrient availability in the soil–plant system (Stevenson and Cole, 1999). The main driving force of these microbe-mediated soil nutrient turnover and transformation processes originates from plant litter and rhizodeposition, which supply energy and nutrients for microbes (Fontaine *et al.*, 2007; Kuzyakov, 2011; Sayer *et al.*, 2011; Zhang *et al.*, 2014). Understanding the C–P trade-off between plants and soil microbes has therefore been of considerable interest over many decades (Richardson and Simpson, 2011).

Physiological and molecular research has revealed that arbuscular mycorrhizal (AM) fungi represent an energetically efficient pathway for the acquisition of plant nutrients from soils (Smith *et al.*, 2011). In a similar fashion to plant roots, the extraradical mycelium of AM fungi has been found to acquire organic N or P with attendant microbes that colonize the surface of AM fungal mycelium or the hyphae that are closely attached to the soil (defined as the hyphosphere) (Hodge and Fitter, 2010; Leigh *et al.*, 2011; Wang *et al.*, 2013; Zhang *et al.*, 2014). The fungal hyphae-associated bacteria are able to stimulate the growth of extraradical mycelium, spore germination, and mycorrhizal formation (Xavier and Germida, 2003; Artursson *et al.*, 2006; Frey-Klett *et al.*, 2007), and accelerate the mineralization of organic N in the presence of AM fungi (Hodge *et al.*, 2001; Leigh *et al.*, 2009; Hodge and Fitter, 2010; Leigh *et al.*, 2011; Herman *et al.*, 2012).

Several studies have suggested that the extraradical hyphae of AM fungi exhibit a high C turnover rate (Staddon *et al.*, 2003; Fitter *et al.*, 2004; Godbold *et al.*, 2006) and these hyphae are able to release a substantial amount of carbohydrates which stimulate bacterial growth and vitality (Filion *et al.*, 1999; Mansfeld-Giese *et al.*, 2002; Toljander *et al.*, 2007) or even change the bacterial community composition under *in vitro* culture conditions (Toljander *et al.*, 2007; Scheublin *et al.*, 2010). The hyphosphere appears to be an environment that is C rich but deficient in available P for many microbes, which may stimulate their activity in mineralizing soil phytate-P and then incorporating the available P into microbial biomass P that is potentially available to AM fungal hyphae (Zhang *et al.*, 2014). P locked in bacterial biomass can be released when C becomes limiting, soils undergo cycles of wetting and drying (Richardson and Simpson, 2011), or during processes of higher tropic-level predation such as bacterial grazing by nematodes (Becquer *et al.*, 2014). The hyphosphere processes through which AM fungal hyphae provide active C derived from plant photosynthesis to microbes for soil P mobilization may be a win–win strategy among plant–AM fungi–fungal-associated bacteria in association with the soil ecosystem. Although there is increasing evidence that biological interactions occur between AM fungi and bacteria in nutrient transformation and turnover in hyphosphere soil, the underlying processes and ecological function are not understood. To date, however, no study has investigated whether fungal-associated bacteria assimilate carbohydrates derived from host plant photosynthesis and are deposited in the hyphosphere via the fungal hyphal exudates in exchange for mobilizing the unavailable nutrients from soil. The objective of the present study was to determine whether PSB that colonize the hyphosphere assimilate carbon from ^13^CO_2_-labeled maize through the extraradical hyphae of AM fungi and thereafter are involved in the mineralization and turnover of organic P in soil.

## Materials and methods

### Soil and microcosms

The soil used in this study was collected from Tai’an, Shandong Province, China and has the following physicochemical properties: pH (soil:H_2_O 1:5) 6.4, organic matter 5.19g kg^−1^, mineral N 7.2mg kg^−1^, Olsen-P [(NaHCO_3_) extractable] 4.9mg kg^−1^, and NH_4_Cl-exchangeable potassium 117.3mg kg^−1^. The soil was air-dried, sieved (2mm), and then irradiated before use to eliminate indigenous microorganisms (10 kGy, ^60^Co γ-rays, Beijing Radiation Application Research Center).

**Fig. 1.**
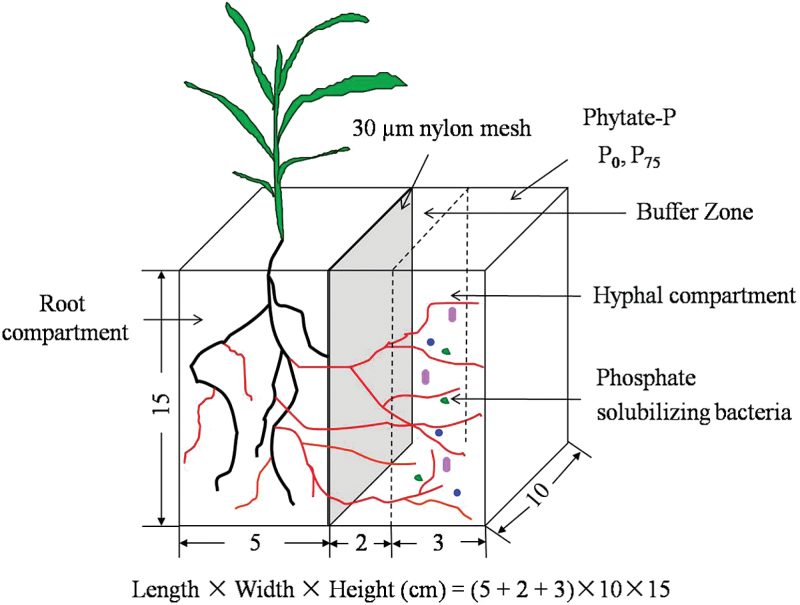
Schematic drawing of the two-compartment cultivation system (rhizobox) used in this study. The rhizoboxes were divided into a root compartment and a hyphal compartment separated by a 30 µm nylon mesh and a buffer zone. Overall dimensions were 10×10×15cm. (This figure is available in colour at *JXB* online.)

The microcosms used were acrylic rhizoboxes constructed to permit the spatial separation of soil zones for root and AM fungal hyphal growth. Each microcosm had two compartments, one for root growth including mycorrhizal structures (root compartment) and a second compartment (buffer zone and hyphal compartment) that was separated from the first compartment by 30 µm nylon mesh through which hyphae (but not roots) could pass (Fig. 1). There was no physical barrier between the buffer zone and the hyphal compartment. The soil density was adjusted to 1.2g cm^−3^ and the method was as follows. First, the volume of each compartment was calculated according to length, width, and height. Secondly, the soil subsamples to be transferred to each compartment were weighed separately based on the volume of the compartment and the target soil density. Thirdly, the soil was transferred very carefully to each compartment to maintain an equal soil density in all compartments. The rhizoboxes received the following amounts of soil: 800g in the root compartment, 320g in the buffer zone, and 480g in the hyphal compartment.

### Host plants, and AM fungal and bacterial inoculants

Maize (*Zea mays* L., cv. Nongda 108) seeds were surface-sterilized with 10% H_2_O_2_ for 10min, thoroughly washed 5–8 times with distilled water, and then germinated on moist filter paper for 2 d at 26 °C in the dark. Two seeds were initially sown in each root compartment and thinned to one seedling after emergence.

The inoculum of *Rhizophagus irregularis* (formerly *Glomus intraradices*, BEG 141, kindly supplied by Professor Vivienne Gianinazzi-Pearson, INRA, France) was propagated on maize and clover and consisted of spores, mycelium, root fragments, and soil. Each mycorrhizal treatment received 15g of inoculum, and every non-mycorrhizal treatment received an equivalent amount of sterilized inoculum in combination with 5ml of filtrate of unsterilized inoculum to provide the same microflora with the exception of the absence of the AM fungus. The filtrate of inoculum was obtained by suspending 30g of unsterilized inoculum in 300ml of sterile water and filtering through five-layer quantitative filter paper (properties similar to Whatman Grade 43), which allowed passage of common soil microbes but efficiently retained spores and hyphae of mycorrhizal fungi. The inoculum was mixed uniformly with the soil of the root compartment before sowing of the germinated seeds.

The PSB *Pseudomonas alcaligenes* M20, *Bacillus megaterium* C4, and *Rahnella aquatilis* HX2 (kindly provided by Professor Sanfeng Chen of the College of Biological Sciences and Associate Professor Yanbin Guo of the College of Resources and Environmental Sciences, China Agricultural University) isolated from rice, maize, and grape rhizospheres, respectively, were used. The three bacterial species were previously tagged with the *gfp* gene encoding green fluorescent protein (GFP) and their capacity to mineralize organic phosphates (Supplementary Fig. S1 at *JXB* online) and colonize AM fungal hyphae (Supplementary Fig. S2) was also previously tested. *Pseudomonas alcaligenes* M20, which was transformed with the pGFP78 plasmid containing the *gfp* gene by electroporation, can release both monoester phosphatase and diester phosphatase to solubilize lecithin or phytate-P (Lv *et al.*, 2005); *B. megaterium* C4, which was labeled with the pGFP4412 plasmid containing the *gfp* gene, can mineralize phytate-P (Zhang *et al.*, 2012); and *R. aquatilis* HX2, which was tagged with the pSMC21 plasmid containing the *gfp* gene, can solubilize both Ca_3_(PO_4_)_2_ and phytate-P (Sun, 2012). All of the PSB strains were grown in liquid Luria–Bertani (LB) medium on an orbital shaker (180rpm) for 24h at 30 °C and then centrifuged at 6000rpm for 10min. The supernatant was discarded and the cells were re-suspended and diluted to 10^8^ CFU ml^−1^ with sterile 155mM NaCl solution. After 30 d of plant growth, equivalent volumes of suspensions of the three bacterial species were mixed together and 10ml of the mixed bacterial suspension was then added to the hyphal compartment in the PSB treatments, whereas an equivalent amount of sterile bacterial suspension was added as a control to the non-inoculated PSB treatments.

### Experimental design

The experiment was set up in a randomized block design with three factors: (i) two different organic P levels; (ii) two AM fungal levels, inoculated with *R. irregularis* or uninoculated; and (iii) two bacterial levels, inoculated with a mixed bacterial suspension or uninoculated. Phytate-P was added (0 or 75mg P kg^−1^ soil) as phytin (TCl, Tokyo, Japan) and was applied only to the hyphal compartment. The experiment was performed in triplicate, and the 24 rhizoboxes were arranged in a randomized block design in the glasshouse. The position of each rhizobox was re-randomized every week. Distilled water was supplied to all of the compartments to maintain the soil moisture level close to field capacity (~20% w/w) during the growth period.

All of the rhizoboxes received basal mineral nutrients which were mixed with the soil uniformly in each compartment at rates of 200mg kg^−1^ N as (NH_4_)_2_SO_4_, 200mg kg^−1^ K as K_2_SO_4_, 50mg kg^−1^ Mg as MgSO_4_·7H_2_O, 5mg kg^−1^ Zn as ZnSO_4_·7H_2_O, 5mg kg^−1^ Mn as MnSO_4_·H_2_O, and 2mg kg^−1^ Cu as CuSO_4_·5H_2_O. In addition, 10mg kg^−1^ P was applied as KH_2_PO_4_ to the root compartment to meet the minimum growth requirement of the plants. In order to enhance the solubilization of phytate-P in soil, (NH_4_)_2_SO_4_ as the N source was supplied to each compartment because AM fungal hyphae release protons to acidify the hyphosphere soil after absorbing ammonium (Wang *et al.*, 2013). Accordingly, the nitrification inhibitor 3,4-dimethylpyrazole phosphate (DMPP; ‘ENTEC Flüssig’ produced by EuroChem Agro GmbH, Mannheim, Germany) was also added, at a rate of 1% (w/w) of the N applied to prevent nitrification of (NH_4_)_2_SO_4_ and maintain a higher NH_4_^+^ concentration in the soil.

### ^13^CO_2_ pulse labeling of plants

#### Treatment selection 

To ensure the transfer of plant-derived C from mycorrhizal hyphae to soil microbes in the hyphosphere, ^13^CO_2_ pulse labeling was repeatedly conducted in the glasshouse over 2 years at the same seedling age and using the same labeling chambers, methods, labeling time period, and harvest time. On the first occasion, four microcosms were established as described above for ^13^C labeling experiments. Both duplicate mycorrhizal and non-mycorrhizal treatments, all with PSB inoculation in the hyphal compartment, were selected to perform ^13^C labeling. Then we found that the ^13^C was enriched in the hyphosphere soil in mycorrhizal treatments but did not appear in non-mycorrhizal treatments (Supplementary Table S1). This suggested that the ^13^C-labeled photosynthates were not enriched in the soil of the hyphal compartment in the absence of AM fungal hyphae. Therefore, in the present experiment, ^13^CO_2_ labeling was applied to the mycorrhizal treatments only together with PSB inoculation in duplicate. Unlabeled mycorrhiza combined with PSB treatment was needed to assess background ^13^C contamination during the two pulse labeling experiments.

#### Labeling chamber and procedure 

**Fig. 2.**
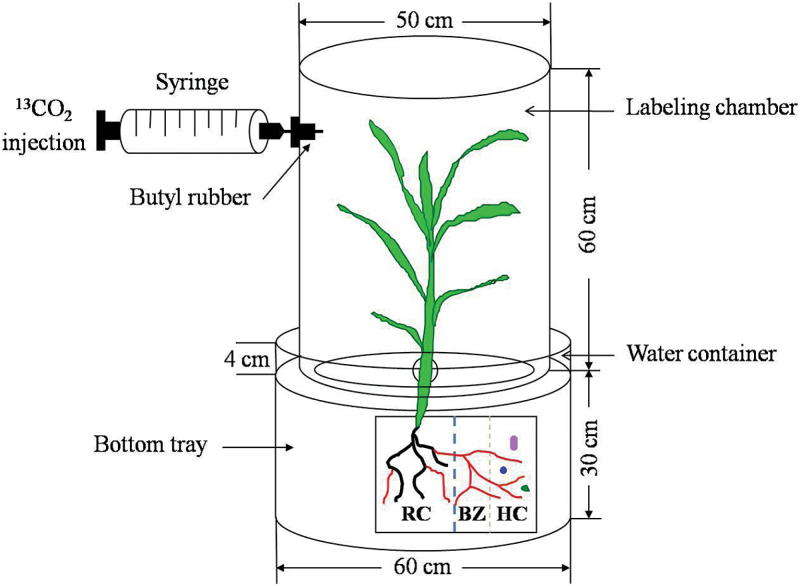
Schematic drawing of pulse labeling of maize plants with ^13^CO_2_. A 75ml aliquot of ^13^CO_2_ (99 atom% ^13^C) was injected every hour from 10:00h to 16:00h (seven times a day) into the top chamber for a total of 5 d. (This figure is available in colour at *JXB* online.)

Fifty days after sowing, the maize plants were subjected to ^13^CO_2_ (99% of ^13^C atom) pulse labeling in an airtight Plexiglas growth chamber (Fig. 2). The chamber (volume 137 dm^3^) had a transparent hood and a bottom tray. The hood was equipped with one hole covered with a septum, the outer edge of the bottom tray was equipped with a water channel to prevent the exchange of ^13^C gas from ambient CO_2_ during the labeling period, and the center of the bottom tray was equipped with three holes fitted around the stem of each plant. The plant shoots protruded through the holes and the joins between stems and holes were sealed with silica gel to prevent exposure of the soil surface to the ^13^C gas. During pulse labeling, the plants were covered with the hood and the fan was fixed in the hood to circulate the air. The hood and bottom were then sealed with water. A 75ml aliquot of ^13^CO_2_ was injected through the septum using a gas-tight syringe every hour from 10:00h to 16:00h. The lid was removed after the last ^13^CO_2_ injection every day, when the CO_2_ concentration in the chamber had decreased to atmospheric level. The labeling was continued for 5 d. The temperature in the glasshouse was controlled at 25–28 °C. To remove the influence of vapor produced by plant evaporation during ^13^CO_2_ labeling on photosynthesis, three trays of CaCl_2_ (100g per tray) were placed in the chamber. The wet CaCl_2_ trays were removed and dried in a forced-air oven at 105 °C for 2h every day after the lid of the chamber was removed in the evening. Thus the CaCl_2_ was dried and re-used repeatedly.

### Harvest and sample analysis

The plants were harvested 8 weeks after sowing. The shoots were dried in a forced-air oven at 70 °C for 48h and weighed. The samples were then ground in a mill before elemental analysis. After the shoot harvest, the rhizoboxes were dismantled and separated into the root and hyphal compartments. To prevent the contamination of the surface soil in the hyphal compartment with exotic bacteria, we removed the top 2cm of soil to reduce the potential influence on hyphosphere soil samples. To obtain a thin slice of the hyphosphere soil, the soil block of the hyphal compartment was placed in an acrylic holder of similar dimensions and then lifted slightly by pushing an acrylic plate (10mm in thickness) underneath. The soil projecting from the holder was then sliced off with a sharp knife. The 10mm thick slice of soil was mixed well and regarded as hyphosphere soil for measuring phosphatase activity, hyphal density, extractable organic P, and microbial biomass P.

#### Analysis of plant samples 

For determination of shoot P concentration, ground plant materials were digested in an H_2_SO_4_–H_2_O_2_ mixture at 370 °C for 2h. The plant P concentration was determined using the standard vanadomolybdate method (Hanson, 1950).

For determination of mycorrhizal colonization, the roots were washed with tap water and cut into 1cm long segments. The root segments were digested in 10% KOH at 90 °C for 60min, rinsed with water, acidified in 2% HCl at 90 °C for 5min, transferred (with no further rinsing) to 0.05% Trypan blue in lactic acid:glycerol (1:1, v/v) solution, and stained at 90°C for 30min. Finally, the roots were decolorized overnight in lactic acid:glycerol (1:1, v/v) solution at room temperature. The intensity of the mycorrhizal colonization (M%) in the roots was determined (Trouvelot *et al.*, 1986) using the MYCOCALC program (http://www.dijon.inra.fr/mychintec/Mycocalc-prg/download.html).

#### Analysis of hyphosphere soil 

Because all types of phosphatases are pH dependent, the phosphatase activity is usually measured at the standard pH to represent their general activity. However, this approach may not correctly reflect the actual phosphatase activity in environmental samples because the pH of the samples differs from the pH used in the assay buffer. We tested the actual acid phosphomonoesterase activity using buffers with pH adjustment according to the measured hyphosphere pH. The acid phosphomonoesterase activity in each hyphosphere soil was assayed using *p*-nitrophenyl phosphate (Sigma, St. Louis, MO, USA) as the substrate according to the method of Joner and Johansen (2000) with slight modifications. Fresh soil was weighed into an Eppendorf tube and incubated for 30min at 30 °C in a water bath with sodium acetate buffer (200mM, adjusted to the respective hyphosphere pH determined for each sample) and reaction substrate. The reaction was terminated by the addition of 0.5M NaOH and the mixture was then centrifuged for 5min at 1500 *g.* The production of *p*-nitrophenol was measured spectrophotometrically at 405nm.

The organic P concentration was measured by the method of Collos and Mornet (1993). Unlike NaOH-EDTA which extracts both soluble and non-soluble organic P that may not be available for soil phosphatase, the Collos and Mornet method extracts organic P with sodium bicarbonate (0.5M NaHCO_3_, pH 8.5); that is, P forms bioavailable for soil phosphatase. In brief, samples of air-dried hyphosphere soil were extracted with 0.5M NaHCO_3_, pH 8.5 (soil:extractant solution 1:20, w/v) at 25 °C for 30min on a reciprocal shaker (180rpm) and then filtered through the quantitative filter paper described above. The soil extract was divided into two parts, one of which was oxidized by acid potassium persulfate in an autoclave for 1h at 121 °C to measure the total P, and the other was used for the determination of inorganic P. The total P and inorganic P concentrations of soil extracts were determined by a modified molybdenum blue colorimetric method (Murphy and Riley, 1962). The extractable organic P was calculated by subtracting the concentration of inorganic P from the concentration of total P.

The microbial biomass phosphorus (MBP) was estimated using the chloroform fumigation–extraction method (Brookes *et al.*, 1982). The weighed fresh hyphosphere soil was fumigated with alcohol-free liquid CHCl_3_ in desiccators. The samples were then extracted with 0.5M NaHCO_3_ (pH 8.5) for 30min on a reciprocal shaker (180rpm) at 25 °C. The extracts were filtered through quantitative filter paper. The Pi concentration in the soil extracts was measured colorimetrically by a modified molybdenum blue method (Murphy and Riley, 1962). MBP was calculated by subtracting the amount of inorganic P (Pi) extracted by 0.5M NaHCO_3_ (pH 8.5) from fresh unfumigated soil from the amount extracted from fumigated soil.

A 5g aliquot of soil in the hyphosphere was used for the estimation of the hyphal length density by a modified membrane filter technique as described previously (Jakobsen *et al.*, 1992). The soil samples were carefully mixed and blended at high speed in a Waring Blender with 50ml of 0.25M sodium oxalate for 30s, and the blended suspension was poured through a 30 μm sieve to retain hyphae. The hyphae were rapidly transferred to a beaker with water and agitated vigorously with a magnetic stirrer at 1000rpm, and then left on the bench for 30s. The 5ml aliquots were pipetted onto 25mm millipore filters (0.45 μm pore size) in a filtration manifold. This procedure was repeated three times. The three filters from each sample were transferred to microscope slides, stained with lactoglycerol–Trypan blue for 5min, and then covered with coverslips. The intersections between blue-stained hyphae and a grid in the eyepiece were counted in 25 fields of view at ×200 magnification.

#### [^13^C]DNA stable isotope probing (SIP) analysis 

##### Determination of carbon isotope ratios of plants and soil

–After the final ^13^C labeling assay, plants were immediately separated into shoots and roots for the labeled and unlabeled treatments. In the case of the hyphosphere soil, labeled and unlabeled samples were collected using the above-mentioned methods. Soil subsamples were frozen in liquid nitrogen immediately and stored at –80 °C until molecular analysis. Other soil subsamples were used for quantification of ^13^C enrichment that is reported as δ^13^C‰ (^13^C:^12^C ratio). Both soil and plant samples were oven-dried at 70 °C, ground, and sieved using an 80 μm mesh. The carbon isotope ratios of these shoot, root, and soil samples were determined using a Delta^Plus^XP mass spectrometer (Thermo Scientiﬁc, Bremen, Germany) coupled with an elemental analyzer (FlashEA 1112; CE Instruments, Wigan, UK) in the continuous ﬂow mode at the Stable Isotope Laboratory of the College of Resources and Environmental Sciences, China Agricultural University, Beijing, China. The elemental analyzer combustion temperature was 1020 °C. The carbon isotopic ratios are reported in the delta notation relative to the V-PDB (Vienna-Pee Dee Belemnite) standard using the following equation:

$$\delta^{\text{13}}\text{C }=\;\left({\text{R}}_{\text{sample}}/{\text{R}}_{\text{standard}}-\text{1}\right)\;\times \text{ 1}000$$

Where δ^13^C is the carbon isotope ratio of the sample in parts per million (‰), and R_sample_ and R_standard_ are the ^13^C/^12^C ratios of the sample and standard, respectively. The SD for the δ^13^C measurements is <0.15‰.

#### DNA extraction, T-RFLP, and clone library construction 

The DNA of hyphosphere soil in labeled mycorrhizal and unlabeled mycorrhizal treatments with PSB inoculation was extracted using the FastDNA SPIN Kit (MP Biomedicals LLC, Santa Ana, CA, USA) following the manufacturer’s instructions, and DNA fractionation was performed by cesium trifluoroacetate (CsTFA) equilibrium density gradient centrifugation (Lueders *et al.*, 2004a). The centrifuged gradients were fractioned from bottom to top into 16 equal fractions. The buoyant density (BD) of DNA in the gradient fractions was determined using a digital refractometer (Reichert AR2000). The DNA fractions were then purified with isopropyl alcohol and 70% ethanol and stored at –20°C for further analysis.

For the T-RFLP (terminal restriction fragment length polymorphism) analysis, DNA fractions of different BDs were amplified using labeled forward primer 27f-FAM (5-carboxyfluorescein) and 907r (Lueders *et al.*, 2004b) under the following reaction conditions: 3min of initial denaturation at 94 °C; 27 cycles of 30s of denaturation at 94 °C, 45s of annealing at 53 °C, and 1min of elongation at 72 °C; and 10min of final elongation at 72 °C. The FAM-labeled PCR products were purified using a TIANgel Midi Purification Kit (Tiangen, Beijing, China) and digested at 37 °C for 5h with *Msp*I (TaKaRa, Shiga, Japan). The digestion products were purified by ethanol precipitation and were then size-separated using an ABI 3730xl DNA analyzer (Applied Biosystems, Waltham, MA, USA).

Unlabeled primers 27f and 907r were used for cloning analysis. PCR amplification was performed using the same reaction conditions as those described above. The PCR products were purified using a TIANgel Midi Purification Kit (Tiangen) and ligated into the pMD19-T vector (TaKaRa) according to the instructions supplied by the manufacturer. The competent cells (TaKaRa) were transformed with the ligation products and spread onto LB agar plates containing ampicillin (100 μg ml^−1^) with X-Gal/IPTG (TaKaRa). White colonies were randomly picked in each clone library and screened directly for inserts by performing colony PCR with the vector primers (M13-47 and RV-M). All of the clones were selected from each clone library and sequenced using an ABI 3730xl DNA analyzer (Applied Biosystems). The raw clone sequences were analyzed with ChromasPro software and then compared with sequences in the NCBI database using the Blast function. Clones that exhibited >98% sequence similarity were selected and a phylogenetic tree was constructed using the Neighbor–Joining algorithm with the MEGA6 software. The sequences obtained in this study were deposited in the GenBank database under the following accession numbers (KF830100–KF830127 and KJ909005–KJ909018).

### Statistical analysis

Statistical analysis was performed with the Statistical Package for Social Sciences version 16.0 (SPSS Inc., Chicago, IL, USA). AM colonization data were arcsine transformed to normalize the distributions before statistical analysis. All statistical analysis data were checked for homogeneity of variances using Levene’s test (*P*>0.05). Different inoculation treatment data were analyzed using one-way ANOVA with organic P level as fixed factor for determining whether differences were significant, and mean values were compared with Duncan’s post-hoc multiple range test at *P*<0.05. Significant differences between zero phytate-P and phytate-P addition treatments for a given inoculation treatment were compared by *t*-test with 95% confidence intervals.

## Results

### AM colonization and external hyphal growth

Very little colonization (<0.35%) was observed in the non-mycorrhizal treatments, the plants were well colonized in the treatments inoculated with *R. irregularis*, and the proportion of root length colonization ranged from 55% to 65% (Table 1).

**Table 1.** Mycorrhizal colonization and hyphal density in the hyphal compartment of maize plants inoculated with *R. irregularis* or uninoculated, and supplied with two organic P levels (0 or 75mg P kg^−1^) and with or without phosphate-solubilizing bacterial inoculation in the hyphosphere

	Root colonization (%)			Hyphal density (m g^−1^ soil)	
	P_0_		P_75_	P_0_	P_75_
Control	0.32±0.03 c A		0.31±0.02 b A	0 c A	0 c A
*R. irregularis*	55.12±2.45 b B		64.57±1.78 a A	12.09±0.13 a B	14.24±0.27 a A
PSB	0.33±0.03 c A		0.29±0.03 b A	0 c A	0 c A
*R. irregularis*+PSB	60.92±0.63 a A		64.83±2.27 a A	9.93±0.31 b B	11.79±0.21 b A
Significance					
Mycorrhiza		***		***	
PSB		NS		***	
Organic P level		**		***	
Mycorrhiza×PSB		NS		***	
Mycorrhiza×organic P level		**		***	
PSB×organic P level		NS		NS	
Mycorrhiza×PSB×organic P level		NS		NS	

Data are presented as the mean (*n*=3) ±SE.

Values followed by different lower case letters in a column or upper case letters in a row are significantly different at the *P*<0.05 level. Significance of treatments and interactions was determined by ANOVA. NS indicates no significant difference, *P< 0.05, **P< 0.01, and ***P<0.001.

No AM fungal hyphae were detected in the hyphal compartment of the non-inoculated treatments, implying that the observed mycorrhizal colonization represents the soil background (Table 1). In contrast, the hyphae proliferated more extensively (9.93–14.24 m g^−1^ soil) into the hyphal compartment (*P*<0.001) in the presence of mycorrhizae with *R. irregularis* independently of phytate-P addition. Compared with the treatments inoculated solely with *R. irregularis*, co-inoculation with PSB and *R. irregularis* significantly reduced the hyphal length density by ~2.2 m g^−1^ soil regardless of phytate-P addition. The addition of phytate-P produced ~1.2 times greater hyphal length density than the P_0_ treatments independently of PSB inoculation.

### Plant biomass and P uptake

Compared with the control, the shoot biomass and P uptake increased on average by 80% and 153%, respectively, when the plants were inoculated with *R. irregularis*. However, PSB inoculation alone did not show this effect regardless of phytate-P addition (Table 2). Compared with inoculation with *R. irregularis*, dual inoculation with PSB and *R. irregularis* did not produce significant effects on the shoot dry weight or P content regardless of phytate-P addition.

**Table 2.** Shoot dry weight and P content of maize with or without *R. irregularis* inoculation, and supplied with two organic P levels (0 or 75mg P kg^−1^) and with or without phosphate-solubilizing bacterial inoculation in the hyphosphere

	Shoot biomass (g per plant)			Shoot P content (mg per plant)		
	P_0_		P_75_	P_0_		P_75_
Control	4.67±0.26 b A		5.09±0.44 b A	3.47±0.23 b A		3.77±0.27 b A
*R. irregularis*	8.29±0.53 a A		9.32±0.12 a A	7.88±0.64 a B		10.52±0.27 a A
PSB	4.52±0.46 b A		5.22±0.18 b A	3.96±0.52 b A		3.77±0.09 b A
*R. irregularis*+PSB	8.61±0.17 a A		9.62±0.45 a A	9.49±0.86 a A		10.99±1.11 a A
Significance						
Mycorrhiza		***			***	
PSB		NS			NS	
Organic P level		NS			*	
Mycorrhiza×PSB		NS			NS	
Mycorrhiza×organic P level		NS			*	
PSB×organic P level		NS			NS	
Mycorrhiza×PSB×organic P level		NS			NS	

Data are presented as the mean (*n*=3) ±SE. Values followed by different lower case letters in a column or upper case letters in a row are significantly different at the *P*<0.05 level. Significance of treatments and interactions was determined by ANOVA. NS indicates no significant difference, *P<0.05, **P<0.01, and *** *P* < 0.001.

The addition of phytate-P significantly increased the P content (from 7.88mg to 10.52mg per plant) of the plants that were inoculated solely with *R. irregularis* (*P*<0.05), but there were no significant differences between the treatments with or without phytate-P addition when the plants were inoculated solely with PSB, dually inoculated with PSB and *R. irregularis*, or control (Table 2).

### Phosphatase activity, soluble organic phosphorus, and microbial biomass phosphorus (MBP) in the hyphal compartment

Acid phosphomonoesterase activity was assayed using buffers with a pH in the measured pH range (pH 6.2–6.4). The acid phosphomonoesterase activities in the hyphal compartment of the soil were ~1.3 or 1.5 times higher after inoculation with *R. irregularis* or dual inoculation with *R. irregularis* and PSB compared with the control and after inoculation with PSB (Table 3). There was no significant difference between the control and the treatment with PSB inoculation only. The addition of phytate-P enhanced the actual acid phosphomonoesterase activities in the presence of mycorrhiza with *R. irregularis*, including sole inoculation and dual inoculation.

**Table 3.** Soil pH and actual acid phosphomonoesterase phosphatase activity in the hyphosphere of maize with or without *R. irregularis* inoculation, and supplied with two organic P levels (0 or 75mg P kg^−1^) and with or without phosphate-solubilizing bacterial inoculation in the hyphosphere

	pH		Actual phosphatase activity (µg *p*-NPP g^−1^ DW soil min^−1^)	
	P_0_	P_75_	P_0_	P_75_
Control	6.39±0.01 a A	6.40±0.02 a A	0.45±0.03 b A	0.47±0.01 b A
*R. irregularis*	6.20±0.01 c A	6.20±0.02 b A	0.64±0.01 a B	0.70±0.01 a A
PSB	6.37±0.01 a A	6.39±0.01 a A	0.49±0.02 b A	0.48±0.04 b A
*R. irregularis*+PSB	6.29±0.03 b A	6.23±0.00 b A	0.60±0.01 a B	0.72±0.03 a A
Significance				
Mycorrhiza	***		***	
PSB	*		NS	
Organic P level	NS		*	
Mycorrhiza×PSB	**		NS	
Mycorrhiza×organic P level	*		*	
PSB×organic P level	NS		NS	
Mycorrhiza×PSB×organic P level	NS		NS	

Data are presented as the mean (*n*=3) ± SE. Values followed by different lower case letters in a column or upper case letters in a row are significantly different at the *P* < 0.05 level. Significance of treatments and interactions was determined by ANOVA. NS indicates no significant difference, *P<0.05, **P<0.01, and *** *P*<0.001.

Compared with the control, the NaHCO_3_-extractable organic P concentrations in the hyphal compartment of the soil decreased by 29% or 42% in the treatments of inoculation with PSB or dual inoculation with PSB and *R. irregularis* in the presence of phytate-P addition, whereas the organic P concentrations decreased by 32% under dual inoculation with PSB and *R. irregularis* treatment in the absence of phytate-P (Table 4).

**Table 4.** NaHCO_3_-extracted organic P and microbial biomass phosphorus (MBP) in the hyphosphere of maize with or without *R. irregularis* inoculation, and supplied with two organic P levels (0 or 75mg P kg^−1^) and with or without phosphate-solubilizing bacterial inoculation in the hyphosphere

	Organic P concentration (mg kg^−1^)			MBP (mg kg^−1^)	
	P_0_		P_75_	P_0_	P_75_
Control	6.36±0.58 a B		8.65±0.38 a A	1.90±0.03 c B	2.45±0.09 c A
*R. irregularis*	5.65±0.62 ab B		7.61±0.55 a A	2.08±0.02 c B	2.59±0.02 c A
PSB	5.45±0.02 ab A		6.12±0.22 b A	2.85±0.07 b B	3.44±0.07 b A
*R. irregularis*+PSB	4.32±0.59 b A		5.05±0.32 b A	4.45±0.10 a B	4.87±0.09 a A
Significance					
Mycorrhiza		**		***	
PSB		***		***	
Organic P level		***		***	
Mycorrhiza×PSB		NS		***	
Mycorrhiza×organic P level		NS		NS	
PSB×organic P level		*		NS	
Mycorrhiza×PSB×organic P level		NS		NS	

Data are presented as the mean (*n*=3) ±SE. Values followed by different lower case letters in a column or upper case letters in a row are significantly different at the *P*<0.05 level. Significance of treatments and interactions was determined by ANOVA. NS indicates no significant difference, * *P*<0.05, ** *P*<0.01, and *** *P*<0.001.

The addition of phytate-P enhanced the MBP concentration in the hyphal compartment of the soil (*P*<0.001). Inoculation with PSB, regardless of whether it was combined with *R. irregularis*, significantly increased the MBP content per kilogram of soil (*P*<0.001) compared with the control or that obtained after inoculation with *R. irregularis* in the presence of both 0 and 75mg kg^−1^ phytate-P (Table 4).

### Assimilation of ^13^C-labeled carbohydrate by active bacteria associated with extraradical hyphae

In unlabeled mycorrhizal treatments, the isotopic signature (δ^13^C) of shoots, roots, and hyphosphere soil was consistent with the atmospheric concentration, suggesting that ^13^C enrichment detected in the hyphosphere soil was not influenced by root exudates. After pulse labeling, the δ^13^C did differ between mycorrhizal and non-mycorrhizal treatments, independently of shoots, roots, and soil in the hyphal compartment (Supplementary Table S1). Similarly, in ^13^CO_2_ labeling experiment 2, the ^13^C enrichment of roots in the labeled treatments was ~100 times higher than in non-labeled treatments. Accordingly, the δ^13^C of hyphosphere soil in the absence of *R. irregularis* was consistent with a natural background of ^13^C, while *R. irregularis* inoculation led to an increase from –22.1‰ to +176.6‰ (Table 5). The isotopic signature (δ^13^C) in the two labeling experiments demonstrates that the shoots, roots, and soil of the hyphal compartment were enriched in ^13^C immediately after pulse labeling. Carbon translocation was especially rapid to the roots and then was transferred to the hyphosphere soil via the extraradical mycelia of *R. irregularis*, suggesting that microbes exploited a ^13^C-rich pool of recent photosynthates.

**Table 5.** Carbon isotope ratios of plant roots and hyphosphere soil after ^13^CO_2_ application to maize inoculated with *R. irregularis* and phosphate-solubilizing bacteria in the hyphal compartment in labeling experiment 2

Labeling status	Treatment	δ^13^C ‰	
		Root	Hyphosphere soil
Labeled samples	M+PSB	2111.6±235.4	176.6±58.0
Unlabeled samples	M+PSB	–21.4±0.4	–22.1±0.3

Data are presented as the mean (*n*=2) ± SE.

**Fig. 3.**
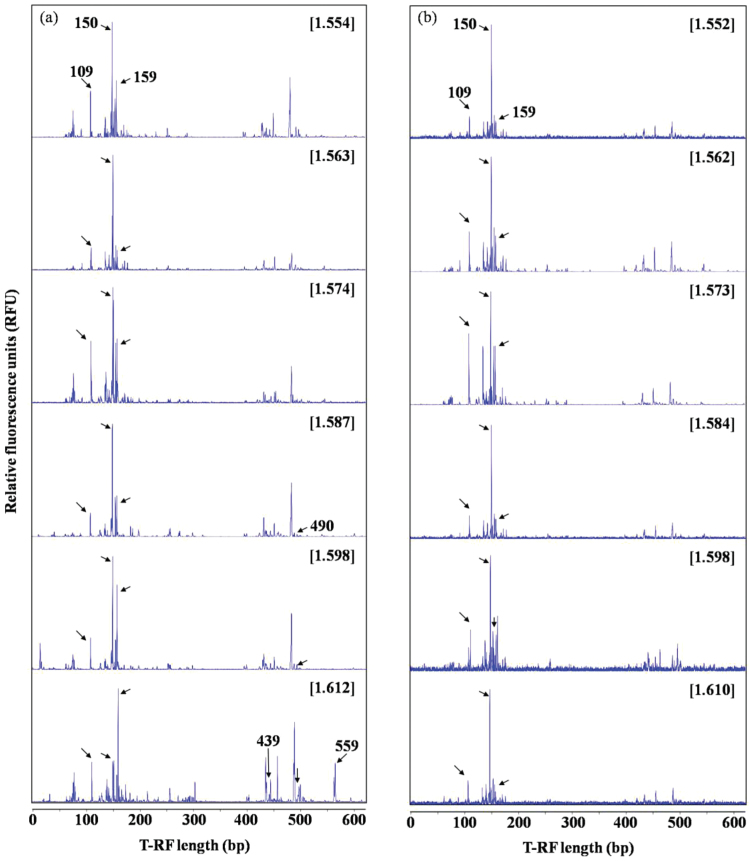
T-RFLP fingerprints of the bacterial 16S rRNA genes retrieved from density-resolved gradient fractions of ^13^C-labeled hyphosphere soil (a) and non-labeled control soil (b) in labeling experiment 2. The CsTFA buoyant densities (g ml^−1^) of the DNA fractions are shown in brackets. The number of base pairs and changes in T-RFs with DNA buoyant density are indicated by arrows.

Maize plants were grown for 5 d with repeated pulses of ^13^CO_2_ (99 atom% ^13^C). DNA isolated from both the ^13^C-labeled and the non-labeled hyphosphere soil was subjected to density fractionation followed by PCR and T-RFLP fingerprinting of bacterial 16S rDNA. These DNA samples were analyzed across a BD gradient from 1.552g ml^−1^ to 1.612g ml^−1^. T-RFLP fingerprints showed that the bacterial community of the non-labeled soil did not change within any of the gradient fractions (Fig. 3b). In the case of the labeled soil, the T-RFLP patterns in ‘light’ DNA fractions (BD ≤1.574g ml^−1^) were similar to those of the unlabeled control soil (Fig. 3a). However, in the ‘heavy’ DNA fractions (BD ≥1.587g ml^−1^), a significant change in the bacterial community was found among the DNA fractions of different BDs. Specifically, the 109bp and 159bp T-RFs were markedly increased in the fractions of BD between 1.587g ml^−1^ and 1.612g ml^−1^. The T-RF with a length of 490bp appeared at a BD of 1.587g ml^−1^ and gradually increased with increasing BD. However, the 150bp T-RF showed a tendency to decrease with an increase in BD and the 439bp and 559bp T-RFs appeared only at a BD of 1.612g ml^−1^. The comparison of the T-RFLP fingerprints between labeled soil and non-labeled soil indicates that the bacterial populations with the characteristics of 109bp, 159bp, and 490bp T-RFs became labeled with ^13^C derived from maize photosynthates.

The ‘heavy’ (BD 1.587g ml^−1^) and ‘light’ (BD 1.563g ml^−1^) DNA fractions were selected to construct clone libraries for bacterial 16S rRNA genes (Table 6). The dominant sequences, which accounted for 45%, were affiliated to Proteobacteria in the ‘heavy’ clone library and the remaining 55% belonged to Actinobacteria, Firmicutes, and uncultured bacteria. In the case of the ‘light’ clone library, Proteobacteria and Cyanobacteria each accounted for 21%, and the other 58% belonged to Actinobacteria, Firmicutes, and uncultured bacteria. Compared with the ‘light’ clone library, the frequency and diversity of the sequences affiliated with the Proteobacteria and Actinobacteria increased in the ‘heavy’ clone library. In contrast, no Cyanobacteria sequences were detected in the ‘heavy’ clone library but they were more abundant in the ‘light’ clone library.

**Table 6.** Phylogenetic affiliations and clone number of bacterial 16S rRNA genes retrieved from ‘heavy’ (BD 1.587g ml^−1^) and ‘light’ (BD 1.563g ml^−1^) DNA fractions of maize hyphosphere soil in labeling experiment 2

Phylogenetic group	Heavy	Light	T-RF (bp)
Alphaproteobacteria			
Bradyrhizobiaceae	4		150, 441
Methylobacteriaceae	1		150
Uncultured	10	3	**150**, 437
Betaproteobacteria			150
Oxalobacteraceae	2		109, 488
Uncultured	7	4	452, 488, 492
Gammaproteobacteria			
Pseudomonadaceae	1		490^*a*^
Uncultured	3	2	82, 141, 186
Actinobacteria			
Streptomycetaceae	5	1	**159**
Nocardioidaceae	1	1	142, 161
Micrococcaceae	1		67
Uncultured	3	1	161
Firmicutes	3	2	145, 153
Cyanobacteria		9	**504**
Uncultured	21	20	82, 95, **113**, **141**, **153**, 183, 287, 603

T-RFs with a relative abundance of >5% in total (105 clones) are indicated in bold.

^*a*^ T-RF represents phosphate-solubilizing bacterium *Pseudomonas alcaligenes* added to the hyphal compartment in this study.

**Fig. 4.**
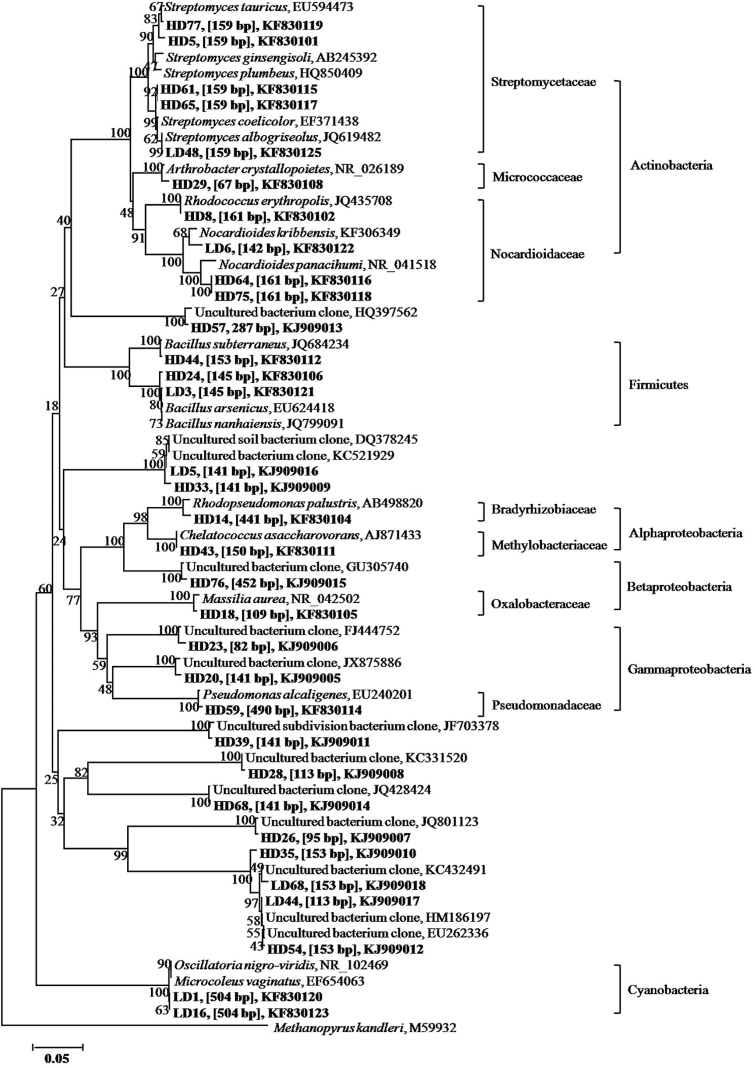
Phylogenetic relationship of bacterial 16S rRNA gene clone sequences derived from ‘heavy’ and ‘light’ DNA fractions of maize hyphosphere soil in labeling experiment 2. Sequences of the HD library represent the ‘heavy’ DNA fraction; those of the LD library represent the ‘light’ DNA fraction. The sequences obtained in this study are shown in bold, the *in silico* T-RF sizes of clone sequences digested with *Msp*I are given in brackets, and the GenBank accession numbers of sequences are indicated. The scale bar represents 5% sequence divergence.

*In silico* analysis of the clone sequences showed that the Alphaproteobacteria group was mainly characterized by 150bp T-RF (Table 6; Fig. 4). One clone of Oxalobacteraceae in the Bateproteobacteria group and one clone of Pseudomonadaceae in the Gammaproteobacteria group showed 100% similarity to *Massilia aurea* and *P. alcaligenes*, and those characterized by T-RFs of 109bp and 490bp were detected only in the ‘heavy’ clone library with a minor abundance (Table 6; Fig. 4). Organisms related to Streptomycetaceae were characterized by a 159bp T-RF and showed higher abundance in the ‘heavy’ clone library. A comparison between T-RFLP fingerprints and the clone library indicates that an increase in the 159bp T-RF in the ‘heavy’ DNA fractions in the ^13^C-labeled hyphosphere soil (Fig. 3a) was consistent with the higher abundance of Streptomycetaceae (Table 6).

## Discussion

### Mineralization and turnover of phytate-P in the hyphosphere

Soil phosphatases are generally classified into acid and alkaline phosphatases based on their optimum pH (Vincent *et al.*, 1992) and into phosphomonoesterases or phosphodiesterases based on the combined bond of the enzyme (Nannipieri *et al.*, 2011). Phytase is one of the groups of phosphomonoesterases that are substrate specific for phytate mineralization and is generally classified into four subgroups (Lim *et al.*, 2007). The overlapping of different groups is obtained after measuring phosphatase using a substrate that contains phosphomonoester bonds in the pH range. For example, purple acid phosphatases are a phosphomonoesterase and a phytase that has maximum activity at acidic pH (Tran *et al.*, 2010). Therefore, we measured the mean activity of phosphomonoesterases that may be involved in the mineralization of phytate in the hyphosphere soil.

Phytate is the dominant form of organic P in soils (Turner *et al.*, 2002). In order to distinguish the added phytate-P from the original soil P pool, we established control treatments that were not inoculated with AM fungi or did not receive phytate-P. We found that the AM fungi led to a positive response in plant biomass even when no additional phytate-P was added (Table 2), suggesting that the extraradical mycelia of *R. irregularis* absorbed P from the original soil P pool and delivered it to maize.

It is found that soil organic P will be overestimated when inorganic polyphosphates are present because polyphosphates do not react with the molybdate reagent during the measurement of inorganic P by molybdate blue colorimetry (Ron Vaz *et al.*, 1993), but they may be hydrolyzed to inorganic P in the processes of oxidization (McKelvie, 2005) and therefore are included in the organic fraction (Haygarth *et al.*, 1998). However, this drawback of the soil organic P measurement method will not influence the results of our present study. First, inorganic polyphosphates in soil occur in bacterial and fungal cells; their concentration is low compared with the soil organic P concentration (Turner *et al.*, 2014) and thus they cannot be included in soil MBP. The highest MBP content in the dual inoculation treatment (Table 4) indicated that the treatment had a higher microbial biomass than the other three treatments. Secondly, the highest microbial biomass should potentially contain more inorganic polyphosphates if inorganic polyphosphates were to exist in the soil sample. We assume that if the soil organic P was to be mineralized with the same mineralization rate in all treatments, the detected organic P concentration should be the highest in the dual inoculation treatment. However, our results were completely opposite (Table 4), suggesting that organic P concentrations in this study were not overestimated, and the mineralization of organic P happened at different rates in different treatments.

Our previous study indicated that AM fungal hyphae release protons as they absorb ammonium and decrease the soil pH in the hyphosphere, which promotes the solubilization of phytate-P and enhances substrate availability of phytate-P to phosphatase (Wang *et al.*, 2013). Therefore, we supplied (NH_4_)_2_SO_4_ as the N source to promote the acidification in hyphosphere soil. Additionally, a nitrification inhibitor named 3,4-dimethylpyrazole phosphate (DMPP) was also added to maintain a higher NH_4_^+^ concentration in the soil. The soil pH was significantly lower in the treatments inoculated with *R. irregularis* compared with the treatments without *R. irregularis* inoculation, and the lower soil pH supported the higher acid phosphomonoesterase activity (Table 3). Because the AM fungus and all of the PSB strains inoculated into the hyphal compartment are able to release phosphomonoesterase and mineralize phytate, we hypothesized that dual inoculation with both *R. irregularis* and PSB can enhance phytate mineralization and increase plant P uptake from phytate by enhancing phosphomonoesterase activities compared with that obtained after inoculation solely with *R. irregularis* or sole treatment with PSB. The results partly support our hypothesis. We found that the dual inoculation and sole *R. irregularis* treatments resulted in higher phosphomonoesterase activities compared with the treatments without *R. irregularis* inoculation (Table 3). The enhanced phosphomonoesterase activities reduced the soil organic P concentrations in the dual inoculation treatments compared with the inoculation with *R. irregularis* (Table 4). However, we did not find any enhancement of P uptake or shoot biomass in the dual inoculation treatment compared with the sole *R. irregularis* treatment (Table 2), but we found that the MBP contents increased in the dual inoculation treatment compared with the sole PSB or sole *R. irregularis* treatments (Table 4) and that the growth of the fungus (hyphal length density) was reduced (Table 1), suggesting that phytate-P mineralized by PSB was not delivered to the plants via the hyphae of *R. irregularis* but was incorporated into the MBP pool. This result is consistent with previous findings that the fungal-associated PSB are more competitive than the AM fungus in acquiring available P and the fungus loses out in the competition that inhibits fungal growth (Zhang *et al.*, 2014).

In mycorrhizal research, the filtrates of inocula that may contain bacterial species and nutrient elements are added to the control treatments to maintain the same microbial flora with the exception of the AM fungus. Phytate-P supply enhanced the organic P concentration only in the control treatment and enhanced the MBP content in all four inoculated treatments. However, the organic P concentration in the control treatment was the highest but the MBP content was the lowest (Table 4). These results suggest that although the AM fungal inocula introduced some bacteria that may have the ability to mineralize phytate-P, their number and effects are markedly lower than the inoculated PSB. In addition, the findings support the aforementioned interpretation that the fungus is involved in the mineralization of phytate-P and then delivers it to the plants in the absence of a PSB competitor, whereas the mineralized phytate-P was preferentially incorporated into the MBP pool under conditions in which a PSB competitor is enriched based on their own advantage.

### Carbon flow in the plant–AM fungi–PSB continuum

AM fungi are obligate biotrophs whose growth relies on carbon from a living host (Smith and Read, 2008). Mycorrhizal fungi have been estimated to receive up to 20% of the plant photosynthetic products, and are maintained only for 5–6 d followed by starting turnover (Staddon *et al.*, 2003). Such a high rate of C turnover is significant even within the context of the global C balance (Szuromi, 2003). However, the pathways in which the photosynthates are consumed at such a rapid rate remain uncertain, although some studies have attributed this consumption to fungal respiration (Johnson *et al.*, 2002, 2005). Recent studies showed that ^13^CO_2_-labeled photosynthates were rapidly transferred to AM fungi, followed by gradual release of the C to their associated microbial groups in the mycorrhizosphere and hyphosphere (Drigo *et al.*, 2010; Kaiser *et al.*, 2015). However, no study has investigated whether any functional bacterial group assimilates photosynthates derived from AM fungal structures and plays a significant role in soil nutrient turnover under soil conditions. In the present study, the C isotope signature of both plant materials (containing shoots and roots) and hyphosphere soil was detected after ^13^CO_2_ was fed to maize plants in the two ^13^CO_2_ labeling experiments (Supplementary Table S1; Table 5), suggesting that photosynthates flow from plant roots to hyphosphere soil via extraradical mycelia of *R. irregularis*. Although the results of bacterial T-RFLP fingerprinting and the clone library were not obtained in labeling experiment 1 due to technical problems, we obtained these data from labeling experiment 2. [^13^C]DNA-SIP analysis reveals that active bacterial groups in the hyphosphere soil incorporated C into their DNA that was sufficiently enriched in ^13^C to allow separation by density gradient centrifugation (Fig. 3). In this study the active bacteria in the hyphosphere were those closely related to Oxalobacteraceae within the Betaproteobacteria, Streptomycetaceae within the Actinobacteria, and Pseudomonadaceae within the Gammaproteobacteria (Table 6). The 109, 159, and 490bp T-RF characteristics for these organisms increased markedly in the ‘heavy’ fractions, but no change occurred in the ‘light’ fractions of the labeled soil (Fig. 3a) or in any of the fractions of the non-labeled soil (Fig. 3b). It would seem that the three groups of bacteria were highly active in assimilating the ^13^C-labeled substrates derived from extraradical mycelia of *R. irregularis*. In the Oxalobacteraceae group, the closest relative to our DNA sequence data is *Massilia aurea* (Fig. 4), which is a strictly aerobic bacterium and known to colonize the roots of many plant species in the rhizosphere (Ofek *et al.*, 2012). *Streptomyces* spp., according to DNA sequence data, were apparently found to be the abundant population in the Streptomycetaceae group, and these organisms are the original source of many antibiotics. In the Pseudomonadaceae group, the DNA sequence data are exactly the same as for *P. alcaligenes* (Fig. 4), the bacterial strain that was inoculated into the hyphal compartment at the start of our experiment and was able to mineralize organic phosphate (Lv *et al.*, 2005).

Although the soil was pre-sterilized, exotic bacteria in the air would have unavoidably contaminated the microcosms because the experiment was conducted in the open. Some environmental factors such as AM fungal inoculum and watering regime may cause bacterial contamination. Therefore, diverse T-RFLP fingerprints were detected in the soil in the hyphal compartment based on the 16S rRNA gene analysis (Figs 3, 4; Table 6).

In our current study, the fungal hyphae-associated bacteria can use two possible pathways to acquire the ^13^C-labeled photosynthates of maize plants via the AM fungal hyphae during the 5 d of labeling. One is from the hyphal exudates released by living hyphae, and the other is through the decay of dead hyphae. Although the processes of C flow from plants to the hyphosphere can be rapid (Johnson *et al.*, 2002, 2005; Kaiser *et al.*, 2015), particularly given that the half-life of some of the terminal hyphae is just a few days (Staddon *et al.*, 2003), the fungal hyphae-associated bacteria are unlikely to be able to assimilate the dead debris of the fungus for the following reasons. First, the ^13^C-SIP method normally uses highly enriched ^13^CO_2_ (99%) and pulse labeling for several days to make the ^13^C-labeled DNA in rhizosphere bacteria detectable (Lu and Conrad, 2005). Secondly, the active cytoplasm is usually retracted to the backbone hyphae by forming septa upon death of the terminal hyphal branches (Bago *et al.*, 1998; Logi *et al.*, 1998), and there is not much ^13^C-labeled food left for the degraders (Jansa *et al.*, 2013). Thirdly, the cell walls of AM fungal hyphae cannot to be degraded rapidly because they are composed of up to 40% of chitin, a carbohydrate that is recalcitrant to decomposition (Zhu and Miller, 2003). Moreover, a weighted mean residence time of chitin-derived products is 49±19 years (Gleixner *et al.*, 2002). Our experiments involved harvesting 5 d after the beginning of ^13^C labeling. In such a short time, the fungal hyphae-associated PSB strains, particularly *P. alcaligenes*, are unlikely to be enriched by ^13^C by degrading the fungal cell wall, indicating that the PSB strain most probably assimilated ^13^C derived from the hyphal exudates. To the best of our knowledge, this study provides the first evidence that a PSB strain colonizing the hyphosphere assimilates photosynthates of the host plant from hyphal exudates of an AM fungus. In addition, the hyphal exudates prime the activity of the PSB, which further accelerates phytate-P turnover in the hyphosphere.

In summary, our findings indicate for the first time that a PSB strain in the hyphosphere, *P. alcaligenes* (Pseudomonadaceae), assimilated the photosynthates fixed by the plant and transferred them to *R. irregularis*, and they were subsequently exuded into the soil and taken up by the PSB. In combination with the AM fungus, the PSB promoted the processes of mineralization and turnover of phytate-P in hyphosphere soil. Thus, while the AM fungus and maize plants had a symbiotic relationship (i.e. they both benefited from the presence of each other), the PSB benefited from the plant and AM fungus but did not provide any reciprocal benefit to either in this experiment. Further studies are needed to link the interaction between AM fungi and the fungal-associated functional microbes to nutrient transformation and turnover in hyphosphere soil.

## Supplementary data

Supplementary data are available at *JXB* online.

Figure S1 An obvious halo zone of phosphate solubilization on National Botanical Research Institute’s phosphate (NBRIP) agar plates produced by *Pseudomonas alcaligenes* M20 (a), *Bacillus megaterium* C4 (b), and *Rahnella aquatilis* HX2 (c) after 4 d growth at 30 °C. 

Figure S2 Phosphate-solubilizing bacteria colonizing the surface of extraradical hyphae of *Rhizophagus irregularis*. (a) *Pseudomonas alcaligenes* M20, (b) *Bacillus megaterium* C4, and (c) *Rahnella aquatilis* HX2. 

Table S1 Carbon isotope ratios of shoots, roots, and hyphosphere soils after ^13^CO_2_ application to maize inoculated with *R. irregularis* or uninoculated, and supplied with phosphate-solubilizing bacteria in the hyphal compartment in labeling experiment 1.

Supplementary Data
