# Supplementary material for: In situ stable isotope probing of phosphate-solubilizing bacteria in the hyphosphere
Source: J Exp Bot. 2016 Jan 21;67(6):1689–701. doi: 10.1093/jxb/erv561 (PMC4783358; doi:10.1093/jxb/erv561)
Supplement: Supplementary Data [file supp_67_6_1689__index.html]

 In situ stable isotope probing of phosphate-solubilizing bacteria in the hyphosphere — In situ stable isotope probing of phosphate-solubilizing bacteria in the hyphosphere — Supplementary Data 

# *In situ* stable isotope probing of phosphate-solubilizing bacteria in the hyphosphere

## Supplementary Data

Data files

- supplementary\_figures\_S1\_S2\_Tables\_S1.pdf - Supplementary Data
