# Supplementary material for: In situ stable isotope probing of phosphate-solubilizing bacteria in the hyphosphere
Source: J Exp Bot. 2016 Jan 21;67(6):1689–701. doi: 10.1093/jxb/erv561 (PMC4783358; doi:10.1093/jxb/erv561)
Supplement: Supplementary Data [file supp_erv561_supplementary_figures_S1_S2_Tables_S1.pdf]

Journal of Experimental Botany

Title: In situ stable isotope probing of phosphorus solubilizing bacteria in the hyphosphere

Authors: Fei Wang[§], Ning Shi[§], Rongfeng Jiang, Fusuo Zhang, Gu Feng*

Supplementary data

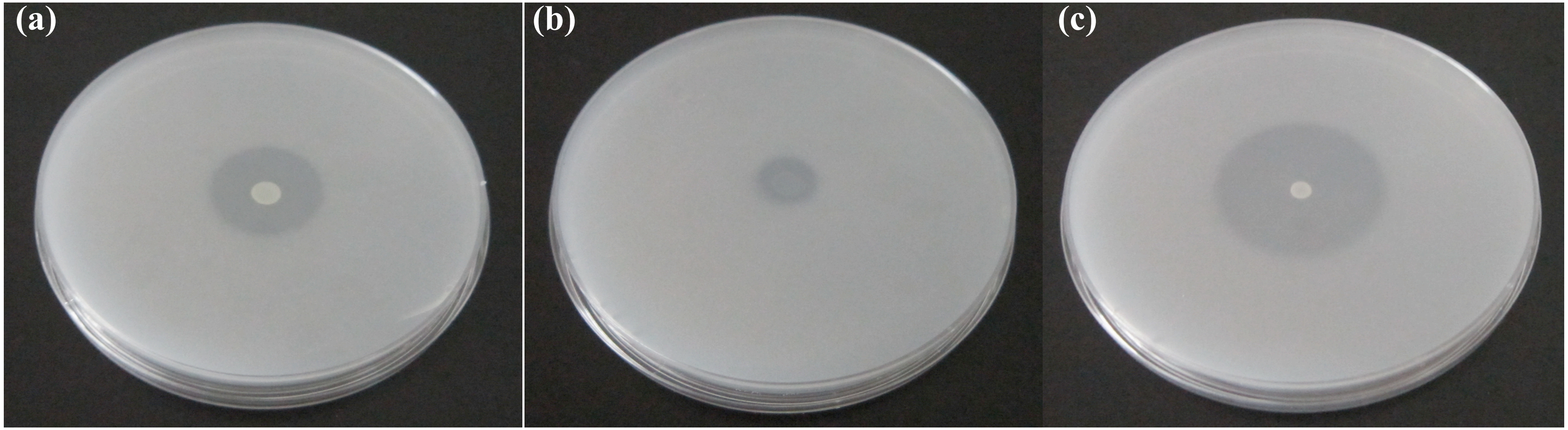

Fig. S1 An obvious halo zone of phosphate solubilization on National Botanical Research Institute's phosphate (NBRIP) agar plates produced by *Pseudomonas alcaligenes* M20 (a), *Bacillus megaterium* C4 (b) and *Rahnella aquatilis* HX2 (c) after 4 d growth at 30°C. NBRIP plate contains (g L^{-1}): glucose 10, $MgCl_2 \cdot 6H_2O$ 5, $MgSO_4 \cdot 7H_2O$ 0.25, KCl 0.2, $(NH_4)_2SO_4$ 0.1, phytate-P 2.

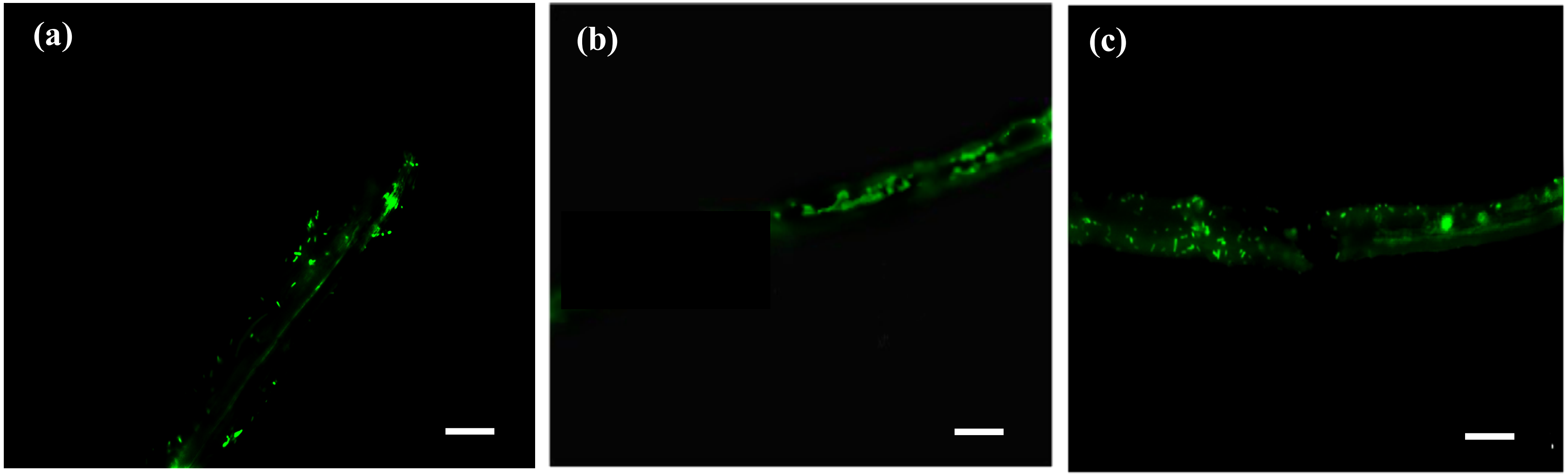

Fig. S2 Phosphate solubilizing bacteria colonizing on the surface of extraradical hyphae of *Rhizophagus intraradices*. (a) *Pseudomonas alcaligenes* M20, (b) *Bacillus megaterium* C4 and (c) *Rahnella aquatilis* HX2. Bars = 10 µm. Images were obtained with an Olympus BX51 fluorescence microscope (Olympus Optical, Tokyo, Japan) and processed with Image Software (Adobe Photoshop CS3).

Table S1 Carbon isotope ratios of shoots, roots and hyphosphere soils after $^{13}CO_2$ application to maize inoculated with *R. irregularis* or uninoculated, and supplied with phosphate solubilizing bacteria in the hyphal compartment in labeling experiment 1

Labeling status	Treatment	$\delta^{13}C$ ‰		
		Shoot	Root	Hyphosphere soil
Labeled samples	NM + PSB	1327.3 ± 138.3	2233.0 ± 326.3	-18.7 ± 3.2
	M + PSB	5212.0 ± 109.0	4624.4 ± 861.6	28.4 ± 0.7
Unlabeled samples	M + PSB	-18.6 ± 0.1	-17.9 ± 0.2	-18.3 ± 2.0

Data are presented as the mean (n = 2) ± standard error (S.E.).
